# dEMBF: A Comprehensive Database of Enzymes of Microalgal Biofuel Feedstock

**DOI:** 10.1371/journal.pone.0146158

**Published:** 2016-01-04

**Authors:** Namrata Misra, Prasanna Kumar Panda, Bikram Kumar Parida, Barada Kanta Mishra

**Affiliations:** 1 Academy of Scientific and Innovative Research, CSIR-Institute of Minerals and Materials Technology, Bhubaneswar, Odisha, India; 2 Bioresources Engineering Department, CSIR-Institute of Minerals and Materials Technology, Bhubaneswar, Odisha, India; National Renewable Energy Lab, UNITED STATES

## Abstract

Microalgae have attracted wide attention as one of the most versatile renewable feedstocks for production of biofuel. To develop genetically engineered high lipid yielding algal strains, a thorough understanding of the lipid biosynthetic pathway and the underpinning enzymes is essential. In this work, we have systematically mined the genomes of fifteen diverse algal species belonging to Chlorophyta, Heterokontophyta, Rhodophyta, and Haptophyta, to identify and annotate the putative enzymes of lipid metabolic pathway. Consequently, we have also developed a database, dEMBF (Database of Enzymes of Microalgal Biofuel Feedstock), which catalogues the complete list of identified enzymes along with their computed annotation details including length, hydrophobicity, amino acid composition, subcellular location, gene ontology, KEGG pathway, orthologous group, Pfam domain, intron-exon organization, transmembrane topology, and secondary/tertiary structural data. Furthermore, to facilitate functional and evolutionary study of these enzymes, a collection of built-in applications for BLAST search, motif identification, sequence and phylogenetic analysis have been seamlessly integrated into the database. dEMBF is the first database that brings together all enzymes responsible for lipid synthesis from available algal genomes, and provides an integrative platform for enzyme inquiry and analysis. This database will be extremely useful for algal biofuel research. It can be accessed at http://bbprof.immt.res.in/embf.

## Introduction

With the irreversible depletion of petroleum resources, renewable biofuels are sustainable alternative to meet the global energy needs. Microalgae as a rich source of lipid, especially triacylglycerols (TAGs) have emerged as a potential biofuel feedstock due to several distinct advantages over other starch-based or lignocellulosic plant species, such as higher photosynthetic efficiency and higher biomass production rate. Besides, microalgae can be grown on non-arable land using wastewater, thus not competing with agri-resources and mitigating CO_2_ emissions efficiently [1, 2]. However, to make biofuel production from microalgae a cost-competitive process, the oil content in oleaginous algae needs to be significantly improved through genetic engineering techniques [3, 4]. It has been proposed that lipid biosynthesis can be increased by over expressing the rate-limiting enzymes of fatty acid biosynthesis pathway, of which acetyl-CoA carboxylase (ACCase) that catalyzes the first committed step of fatty acid synthesis viz., conversion of acetyl CoA to malonyl CoA plays a pivotal role [5, 6]. In addition, overexpression of the acyltransferases enzymes catalyzing the main regulatory steps involved in TAG biosynthesis, widely known as the Kennedy pathway, have also been determined as a potential approach to boost oil accumulation. For instance, overexpression of a type 2 diacylglycerol acyltransferases (DGAT) enzyme in the diatom *Phaeodactylum tricornutum* resulted in 35% increase in TAG content [7]. In another study, co-overexpression of multiple genes of the Kennedy pathway including glycerol-3 phosphate acyltransferase (GPAT), lysophosphatidyl acyltransferase (LPAT), phosphatidic acid phosphatase (PAP), diacylglycerol acyltransferase, glycerol-3 phosphate dehydrogenase (GPDH) and phospholipid:diacylglycerol acyltransferase (PDAT) in *Chlorella minutissima* resulted in a two-fold increase of TAG content [8]. Introduction of diacylglycerol acyltransferase 2 gene from *Brassica napus* to *Chlamydomonas reinhardtii* has also resulted in enhanced lipid production [9]. Together these studies indicate that understanding the regulation of microalgal lipid metabolism is absolutely essential for developing engineered microalgae with enhanced lipid production capabilities. [10]. While algal sequence data from genome assembly projects is rapidly increasing, the generated annotation for predicted sequences are usually limited and includes only user-defined function prediction with no detailed pathway, structure or genome-context information [11]. This limits our understanding of the overall lipid biosynthetic pathway in microalgae [12]. On contrary, the genes and enzymes involved in plant lipid biosynthetic pathway have been characterized extensively [13], and a number of biomass-related enzyme databases are also available to promote the development of transgenic biofuel crops [14–17]. Considering the importance of microalgae biofuel, paucity of information on algal lipid biosynthesis and unavailability of dedicated databases on enzymes underpinning the process, the present study was performed to identify a total of 289 enzymes responsible for lipid accumulation in fifteen sequenced microalgal species by using available homologous sequences from the model plant species, *Arabidopsis thaliana*. Functional annotation of the putative enzymes has also been improved by employing several bioinformatic tools to study metabolic pathways, ontology, subcellular location, secondary and tertiary structure, biophysical properties, cellular processes and protein family information. Furthermore, the emanated data are made publicly accessible through an open-access web-based database, dEMBF (database of Enzymes of Microalgal Biofuel Feedstock, http://bbprof.immt.res.in/embf). dEMBF is the first integrative platform that provides a complete list of enzymes putatively involved in lipid biosynthesis in microalgae. This database will certainly provide a roadmap for experimental as well as computational studies leading to identification of orthologous lipid synthesis enzymes in newly sequenced algal species and facilitate further R&D research aimed at attaining a sustainable and cost-effective biofuel production from microalgae.

## Materials and Methods

### Data sources

We analyzed a total of fifteen algal genomes belonging to diverse phylogenetic groups, namely Chlorophyta (*Chlorella variabilis*, *Chlamydomonas reinhardtii*, *Volvox carteri*, *Ostreococcus lucimarinus*, *Ostreococcus tauri*, *Micromonas pusilla CCMP1545*, *Micromonas sp*. *RCC299*, and *Bathycoccus prasinos*), Heterokontophyta (*Thalassiosira pseudonana*, *Phaeodactylum tricornutum*, *Ectocarpus siliculosus*, *Aureococcus anophagefferens*, *Nannochloropsis gaditana*), Rhodophyta (*Cyanidioschyzon merolae*) and Haptophyta (*Emiliania huxleyi*). Proteomic sequences were retrieved mainly from the Phytozome (http://www.phtoozome.net) or from dedicated genome project websites for individual targeted species.

### Enzyme prediction

The genome databases were queried by both keywords and sequence similarity BLASTp [18] search (E-value < 1e-5) using sequences of enzymes that are known to be involved in neutral lipid synthesis in Arabidopsis (Table 1). Subsequently, the successful hits were mapped to UniProt ID [19], Enzyme commission (EC) number, Cluster of Orthologous groups (KOG) using KOGnitor [20], OrthoMCL [21] and Gene Ontology (GO) terms [22] using AmiGO [23], to remove any false positives. In addition, Pfam [24] was also employed to ensure that each candidate sequence shared the domain of the enzyme family to which it belongs. Finally, a complete set of 316 enzymes was collected from the studied algal species including Arabidopsis, for further detailed analysis of functional annotations as discussed below.

**Table 1:** List of enzymes involved in lipid biosynthesis in Arabidopsis that were used as query sequences for BLASTp sequence similarity searches on algal genomes. All sequences were retrieved from the Arabidopsis acyl-lipid metabolism website (http://aralip.plantbiology.msu.edu) See Reference [13].

Enzyme description	Enzyme name	EC No	UniProt ID
**Fatty acid biosynthesis**			
Homomeric Acetyl-CoA carboxylase	Homomeric ACCase	6.4.1.2	F4I1L3
Acetyl-CoA carboxylase alpha-carboxyltransferase	alpha-CT	6.4.1.2	Q9LD43
Acetyl-CoA carboxylase beta-carboxyltransferase	beta-CT	6.4.1.2	P56765
Biotin carboxylase	BC	6.3.4.14	O04983
Biotin carboxyl carrier protein	BCCP	6.4.1.2	Q9LLC1, Q42533
Malonyl-CoA-ACP Malonyltransacylase	MCMT/MCAT/FabD	2.3.1.39	Q8RU07
beta-ketoacyl-ACP Synthase I	KAS I/FabF	2.3.1.179	P52410
beta-ketoacyl-ACP Synthase II	KAS II/FabF	2.3.1.179	Q9C9P4
beta-ketoacyl-ACP Synthase III	KAS III/FabH	2.3.1.180	P49243
3-ketoacyl-ACP Reductase	KAR/FabG	1.1.1.100	P33207
3-hydroxyacyl-ACP Dehydratase	HAD/FabZ/FabA	4.2.1.59	Q9LX13, Q9SIE3
Enoyl-ACP Reductase	ENR/FabI	1.3.1.9	Q9SLA8
**Triacylglycerol (TAG) assembly**			
NAD-dependent Glycerol-3-phosphate dehydrogenase	GPDH	1.1.1.8	Q9SCX9, Q949Q0
Glycerol-3-phosphate acyltransferase	GPAT	2.3.1.15	Q8GWG0, Q43307
Lysophosphatidyl acyltransferase	LPAT/LPAAT	2.3.1.51	Q8GXU8, Q8L4Y2, Q8LG50, Q9LHN4, Q9SYC8
Phosphatidate phosphatase	PAP/PP	3.1.3.4	Q9FMN2, Q9SF47
Diacylglycerol acyltransferase Type 1	DGAT1	2.3.1.20	Q9SLD2
Diacylglycerol acyltransferase Type 2	DGAT2	2.3.1.20	Q9ASU1

### Functional annotations

#### Physico-chemical properties

The total number of amino acids, molecular weight, isoelectric point (pI), percentage of acidic/ basic amino acids, aliphatic index as well as GRAVY index was calculated using the Expasy’s ProtParam server [25]. Hydropathy plot was generated using the BioEdit [26] software.

#### Secondary structure prediction

To predict secondary structure (the percentage of residues in helices, extended strands, and random coils) and transmembrane helix domains, MINNOU [27] and TMHMM [28] were used, respectively.

#### Subcellular location

Several prediction tools such as TargetP [29], ChloroP [30], Predotar [31], and WolfPsort [32] were employed for determining subcellular location.

#### Gene structure analysis

The exon-intron organizations of genes encoding the enzymes were determined by GeneWise program [33] through comparison of predicted coding sequence with corresponding genomic sequence.

#### Homology modeling of 3D structures

As no crystal structures of the predicted enzymes for microalgal species was found in the Protein Data Bank [34], we tried to model their 3D structure using MaxMod program [35]. Templates were selected based on crystal structures having more than 30% sequence identity. Ramachandran plot of the developed models were generated using the Procheck [36] program.

### Database architecture

The dEMBF database runs on an Apache server (v. 2.2.17), where PHP v 5.3.5 was used for server side scripting while Java Script, AJAX, XHTML and CSS were used for client side scripting. Data was stored in a relational format using MySQL v 5.0.7 as the backend database, following basic normalization rules in order to reduce data redundancy and increase database efficiency.

## Results

### Annotation details of enzymes in dEMBF

**Fig 1:**
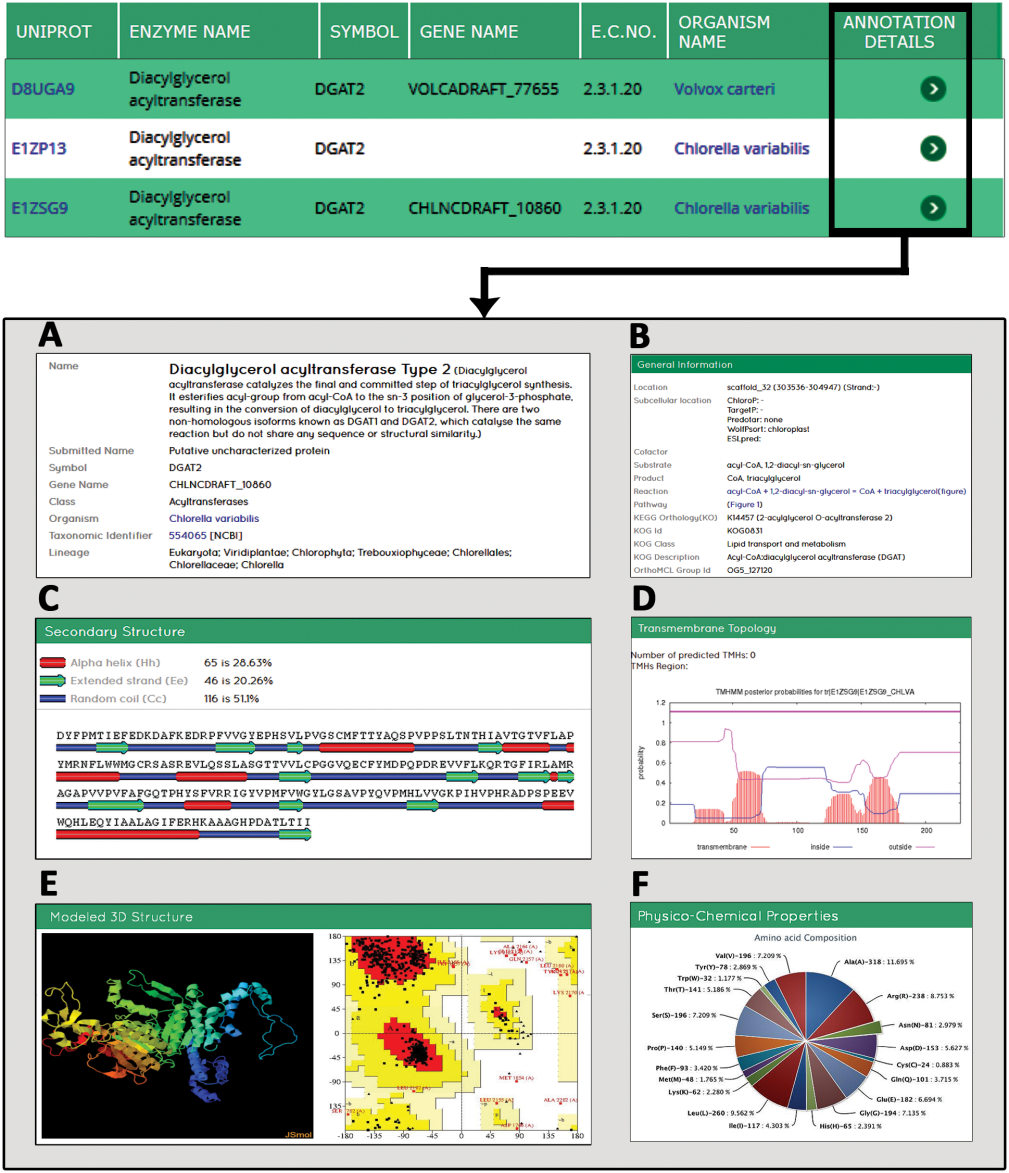
Demonstration of a typical example of annotation details for a lipid biosynthetic enzyme in dEMBF. By clicking on “Annotation details” tab, user will be navigated to a page displaying various information of an enzyme. (A) This field shows enzyme name, symbol, gene name, class, organism, taxonomic identifier and lineage details with hyperlinks to original sources. (B) The general information includes details on chromosomal location, subcellular location, reaction, pathway along with KOG and OrthoMCL ID. (C) An example of secondary structure determined by MINNOU tool. (D) An example of transmembrane topology predicted by TMHMM. (E) Modeled 3D structure and Ramachandran plot is provided. (F) Schematic representation of amino acid composition of an enzyme computed using ProtParam. Please note that not all fields are shown.

The annotation detail page of dEMBF (Fig 1) displays multiple sequence and structural properties of the enzymes that has either been extracted manually from public resources or has been computed using a plethora of bioinformatics tools as described in the Methods section. Each sequence is annotated with information like symbol, gene name, enzyme class, organism, taxonomic identifier which is linked to NCBI taxonomic browser and organism lineage. The general information section furnishes information on chromosomal location, subcellular location, reaction, KEGG pathway, KEGG ortholog (KO) and KOG details. Similarly, the page also contains other predicted protein features such as gene ontology, physico-chemical properties, schematic representation of conserved domain, secondary structure, transmembrane topology, modeled 3D structure, intron-exon organization, amino acid and nucleotide sequences in fasta format, and cross references to external protein and gene databases. Particularly, the modeled 3D structure that has been built using homology modeling protocol along with details of the template employed and target-template alignment will be useful for users to study the structural conformation of enzymes in detail. To facilitate dynamic visualization of developed protein 3D conformation, JSmol applet (http://www.jmol.org) has been integrated. Furthermore, pre-generated Ramachandran plots for each modeled structure can be viewed using the “View Ramachandran plot” option.

### Web-interface of dEMBF

**Fig 2:**
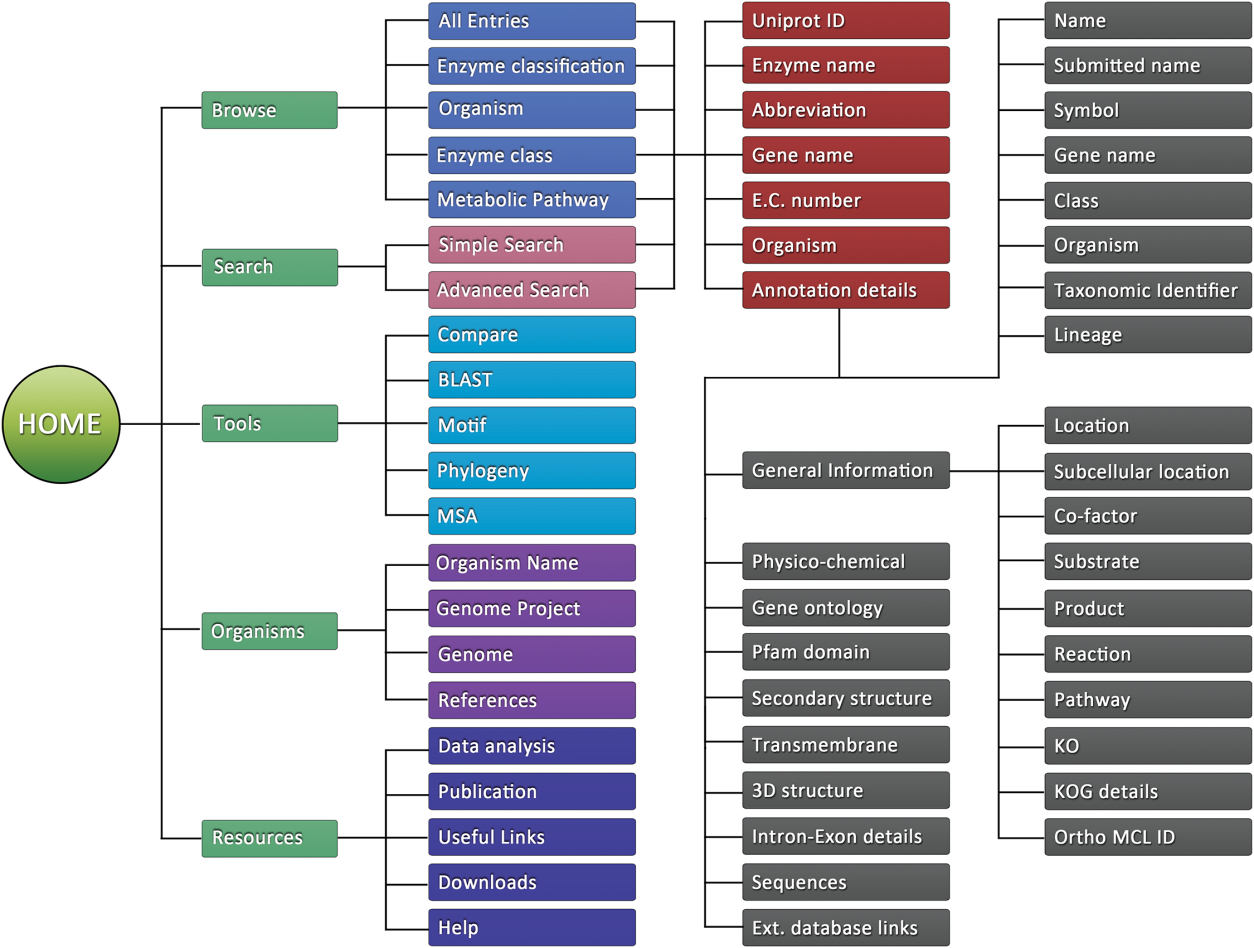
A schematic representation of architecture of dEMBF.

The dEMBF database comprises of six major web interfaces, namely “Home”, “Browse”, “Search”, “Tools”, “Organisms” and “Resources”. A schematic overview of dEMBF architecture is shown in Fig 2.

#### Home

**Fig 3:**
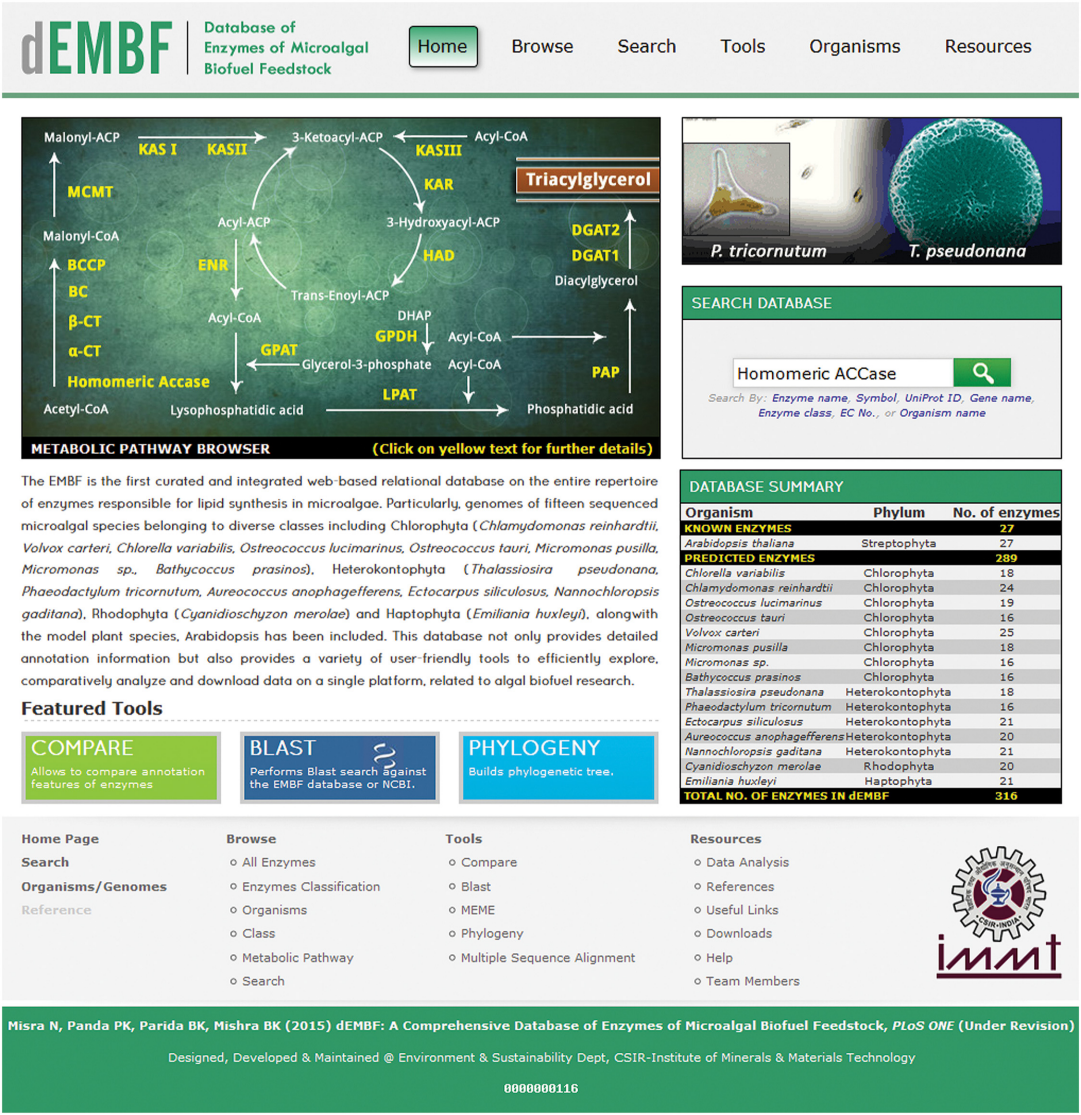
The dEMBF homepage displaying the different options available for the user. The major web interfaces of dEMBF, namely Browse, Search, Tools, Organisms and Resources are at the top right of the page. The “Metabolic Pathway Browser” is provided where users can select any enzymes (yellow color) of the lipid biosynthetic pathway to retrieve its detailed information. The “Database Summary” lists the total number of lipid biosynthetic enzymes currently present in dEMBF. An easy-to-use search field with multiple search criteria including enzyme name, symbol, UniProt ID, gene name, enzyme class, EC number, and organism name is also provided. Links to some of the important tools of dEMBF such as “Compare”, “BLAST” and “Phylogeny” is available in the homepage.

The home page (Fig 3) contains a brief introduction to dEMBF and a site map detailing the outline of the database. Various convenient utilities are also available in the homepage to view and retrieve data from dEMBF. For instance with the “Search database” option, users can search for enzyme by name, symbol, UniProt Id, gene name, enzyme class, EC number, or organism name, directly from the homepage of database using auto complete text fields. Likewise, the “Metabolic Pathway Browser” greatly facilities users to browse detailed information of any particular enzyme by just clicking on the enzyme name that has been manually mapped onto the lipid biosynthetic pathway. The “Database Summary” provides a complete list of the total number of lipid biosynthetic enzymes in various algal species currently present in dEMBF. Links to some of the important tools of dEMBF such as “BLAST”, “Compare” and “Phylogeny”, is also provided.

#### Browse

**Fig 4:**
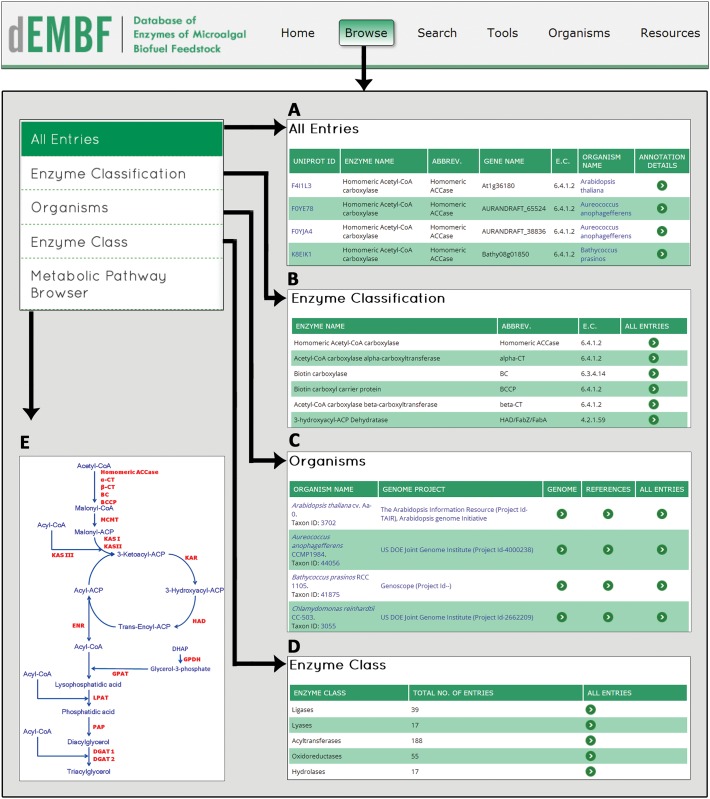
Screenshots of the dEMBF browse interface. A user can browse the database using five different browsing options including (A) Browse by “All Entries”. (B) Browse by “Enzyme Classification”. (C) Browse by “Organism”. (D) Browse by “Enzyme Class”. (E) “Metabolic Pathway Browser”.

A number of browsing options are provided in dEMBF to allow users to navigate by specific criteria, such as selecting browse by “All Entries” for retrieving all enzymes present in the database or browse by “Enzyme Classification”, “Organism”, and “Enzyme Class”, for specific enzymes of interest (Fig 4). On clicking the “Browse” option, user will be redirected to a page displaying all enzymes along with their respective accession ID, abbreviation, gene name, EC number, organism name and annotation details. The “Annotation details”, option provides comprehensive sequence and structural properties of an enzyme (Fig 1), as discussed in the “Annotation details of enzymes in dEMBF” section of results. In addition, the “Metabolic Pathway Browser” is a dynamic browsing interface where the lipid biosynthesis enzymes have been linked to its information details.

#### Search

**Fig 5:**
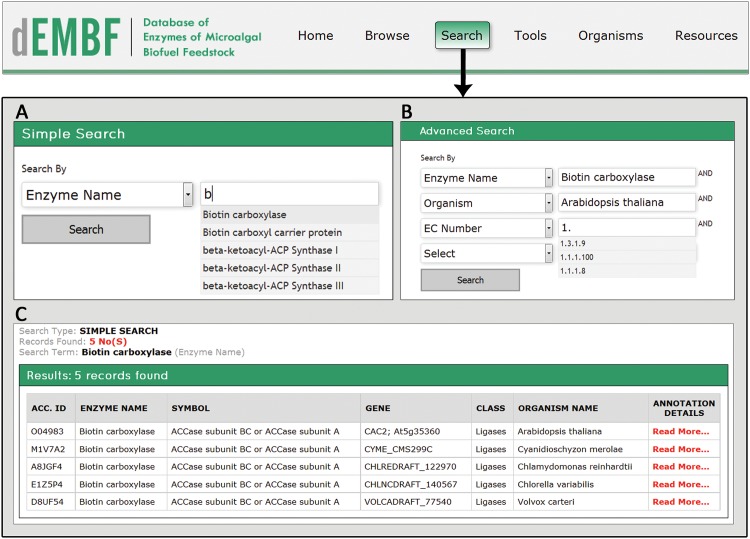
Screenshots of the dEMBF search interface. (A) The “Simple Search” provides search queries for the enzyme name, symbol, gene name, organism, enzyme class, EC number, KOG ID, Gene Ontology, Pfam ID, UniProt ID and gene ID. (B) The “Advanced Search” allows users to input multiple search queries simultaneously to retrieve specific enzymes of interest. (C) An example of a simple search result queried by the enzyme name, biotin carboxylase.

The “Search” function permits users to perform a simple search and advanced search in the database (Fig 5). The “Simple Search” option provides search queries for the enzyme name, symbol, gene name, organism, enzyme class, EC number, KOG ID, Gene Ontology, Pfam ID, UniProt ID and gene ID. The “Advanced Search” allows users to combine multiple search criteria in order to locate specific enzymes of interest more precisely.

#### Tools

A number of web-based tools have been integrated in dEMBF to facilitate further analysis of the enzymes. A brief description of these tools is as follows:

#### BLAST

**Fig 6:**
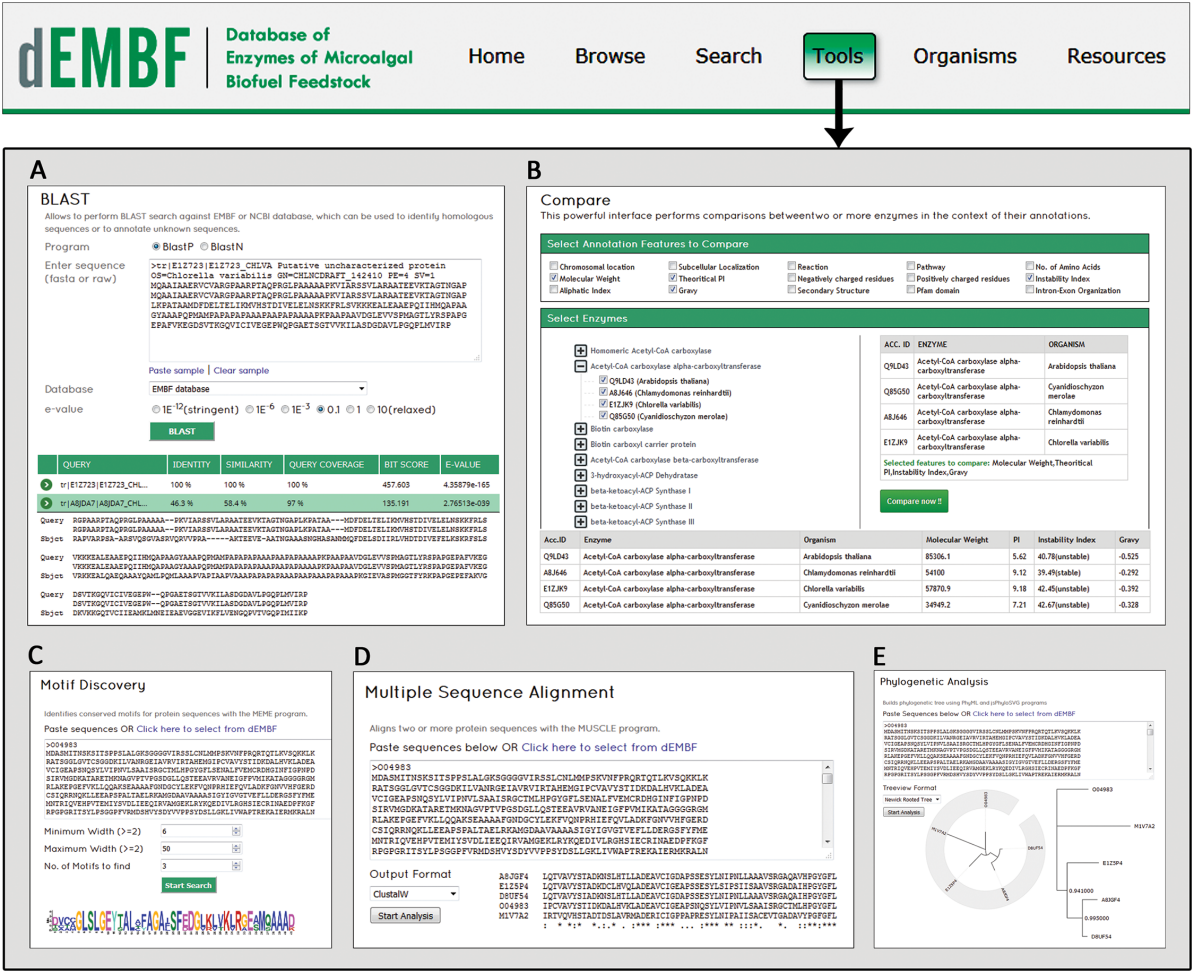
Screenshots of dEMBF analysis tools displaying their query pages and resulting outputs. (A) “BLAST” tool allow users to perform similarity search for protein or nucleotide sequences against NCBI, dEMBF database or against individual enzymes. A wide range of E-values are provided to control search sensitivity. BLAST results are sorted by percentage of identity, similarity, query coverage, bit score and E-value. (B) “Compare” tool to perform comparative analysis of enzyme between one or multiple algal species. Users can select various clickable annotation feature alongwith enzyme name and corresponding organisms between which comparisons is to be carried out. (C) “Motif” tool to identify conserved motifs in query sequences using the integrated MEME program. (D) “MSA” tool to align two or more protein sequences with the MUSCLE program. (E) “Phylogeny” tool to construct phylogenetic tree (Newick rooted tree or Circular tree) using PhyML and jsPhyloSVG.

The standalone NCBI’s BLAST software was integrated as a part of the dEMBF tools. Users can perform a BLAST search of a query sequence either against the entire dEMBF database or against each individual enzyme to identify homologous sequences (Fig 6a). A wide range of E-values are available to control search sensitivity. The BLAST results are displayed on the same page in a tabular format sorted by percentage of identity, similarity, query coverage, bit score and E-value. This interface is particularly useful for users to annotate the function of an unknown sequence.

#### Compare

The “Compare” tool (Fig 6b) allows user to perform comparative analysis of enzymes between one or multiple algal species. User has to select at least two enzymes from the same or different organism alongwith the annotation features based on which the comparison will be carried out. The results are displayed in a condensed tabular format.

#### Motif

Users can predict conserved motifs using the MEME [37] program integrated in dEMBF (Fig 6c). On submission of protein sequences, the database will redirect the query to MEME and after the completion of job, results in various pre-defined formats are made available for download.

#### MSA and Phylogeny

In addition to above tools, both multiple sequence alignment (Fig 6d) and phylogenetic (Fig 6e) tools are also provided in the “Tools” page of dEMBF. Alignment of two or more protein sequences is done by MUSCLE [38] and newick trees are built using PhyML [39] and jsPhyloSVG [40], the later being a java-independent function for viewing phylogenetic tree files online.

#### Organisms

**Fig 7:**
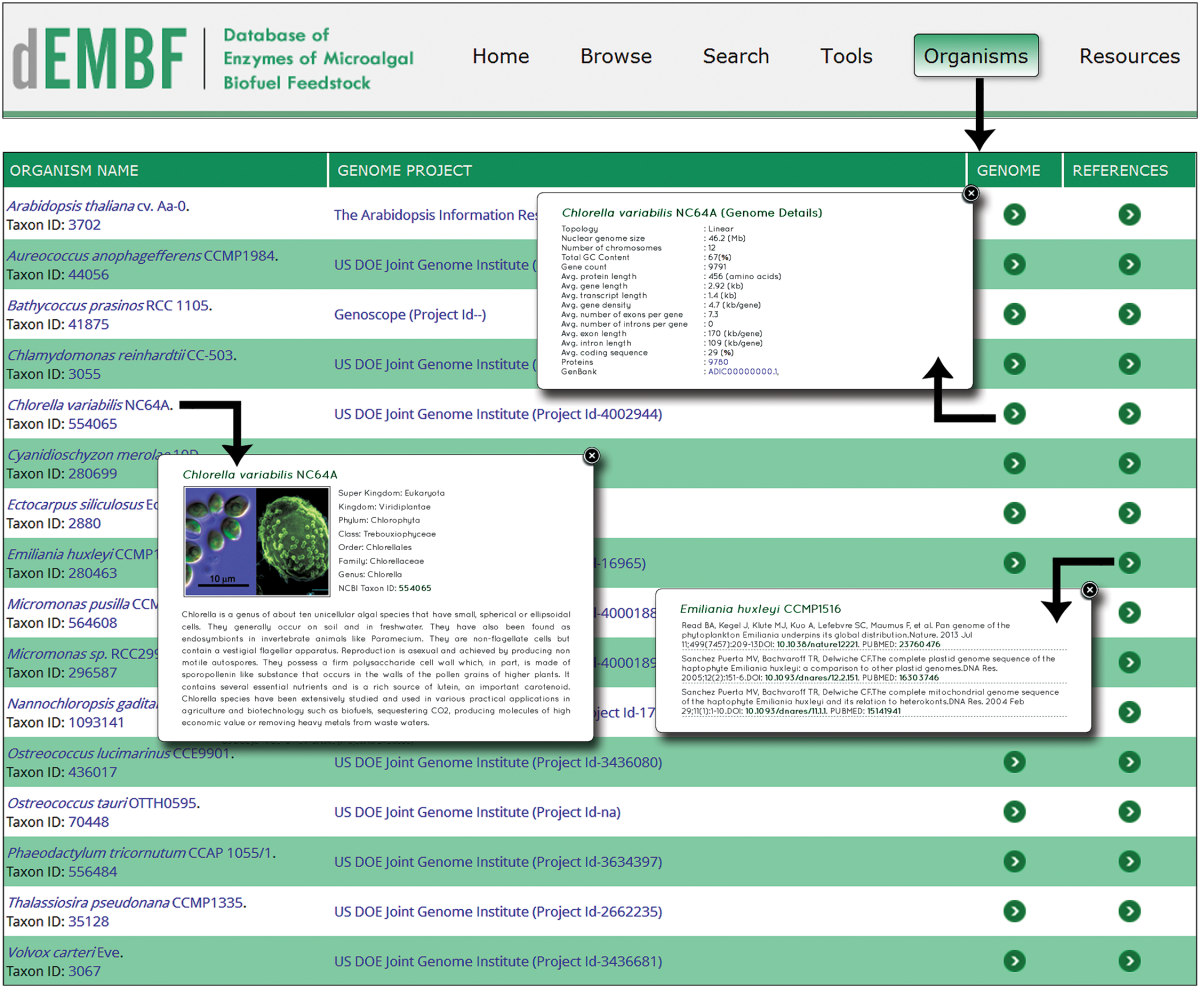
Screenshot of the dEMBF organism page. This page shows the list of algal species analysed in this study alongwith information on their taxonomic lineage, genome project, genome details and references.

The “Organism” page (Fig 7) displays the list of sequenced genomes analyzed in dEMBF, which comprises of fifteen microalgal species alongwith Arabidopsis as the reference plant species. This page includes the name of the organism, corresponding genome project database, genome details and related references, where each of the above fields are linked to further detailed information.

#### Resources

**Fig 8:**
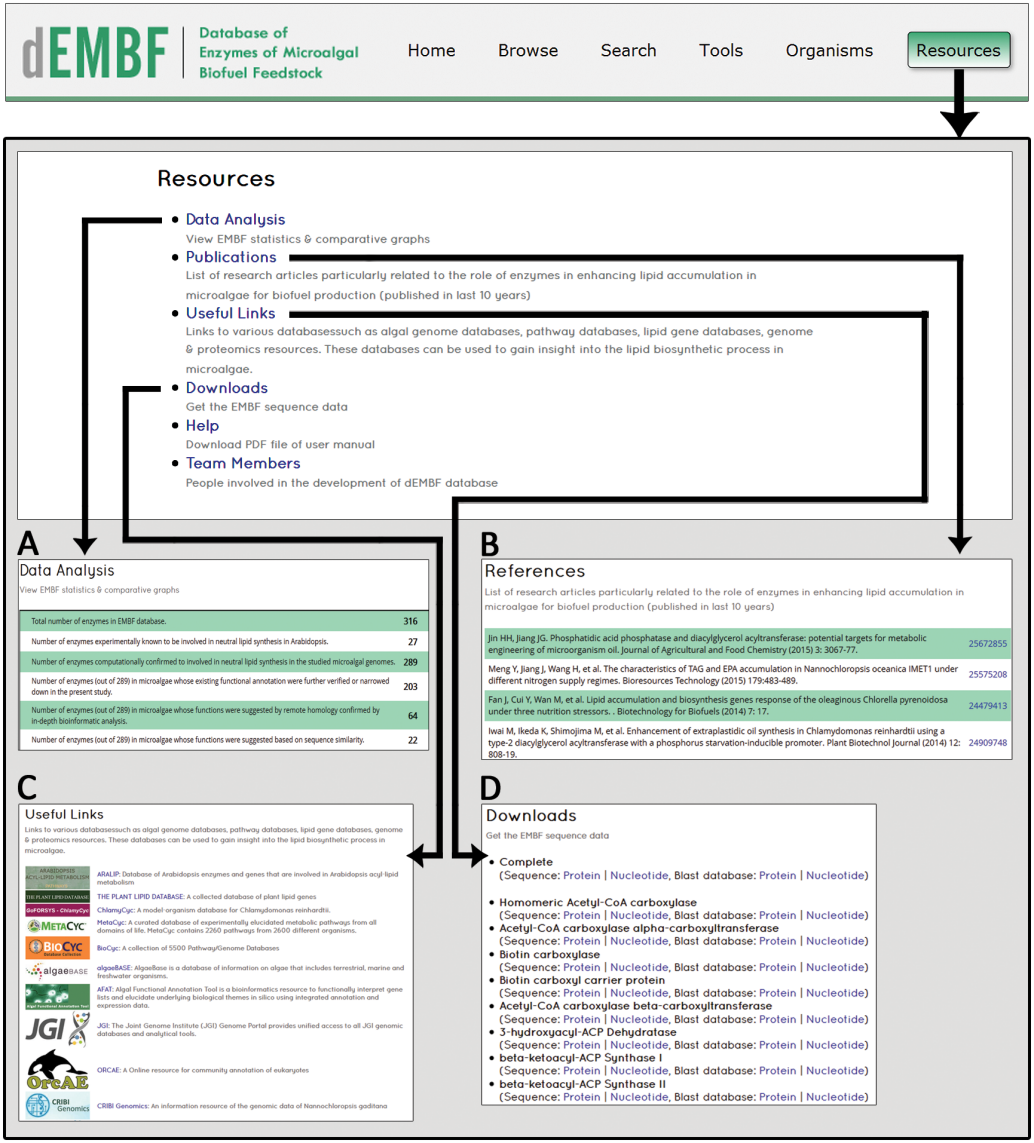
Screenshots of the dEMBF resource page. (A) “Data Analysis” displays a statistical overview of the data present in dEMBF. (B) “Publication” provides a list of research articles on algal lipid biosynthesis with hyperlinks to PubMed database. (C) “Useful Links” tab presents hyperlinks to other important bioinformatic resources such as algal genome project databases (JGI, ORCAE, CRIBI Genomics), Arabidopsis lipid gene database, metabolic pathway databases (KEGG, MetaCyc, BioCyc, ChlamyCyc, and AFAT). (D) “Download” tab allow users to export a copy of the entire dEMBF data.

Given below are brief descriptions of the various utilities, available in the “Resources” page of dEMBF:

Data analysis: A statistical overview of the data present in dEMBF is provided (Fig 8a).
Publications: Recent research articles on algal lipid biosynthesis pathway have been compiled alongwith hyperlinks to PubMed for user references (Fig 8b).
Useful Link*s*: External database links are provided to other bioinformatics resources such as algal genome project databases, Arabidopsis lipid gene database, metabolic pathway databases and protein databases (Fig 8c).
Downloads: All protein and nucleotide sequences present in the dEMBF are available for download from “Download” page of Resources (Fig 8d).
Help: A detailed description on the use of various features of dEMBF database is provided in a Help file.

## Discussion

**Fig 9:**
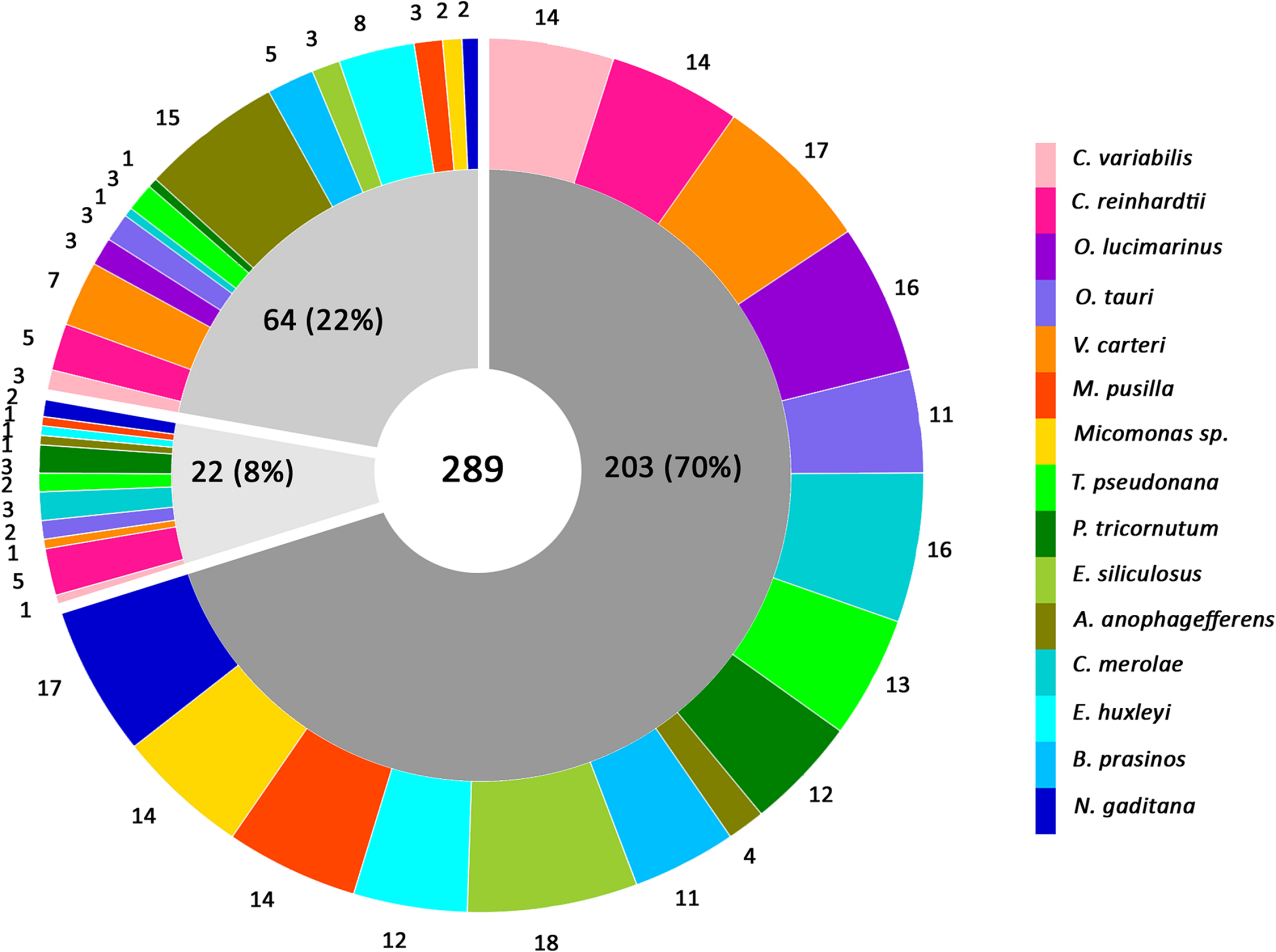
Chart showing the annotations of 289 enzymes present in dEMBF that are putatively involved in lipid biosynthesis in fifteen algal species. The dark grey sector indicates the total number of enzymes with functional annotations available from JGI database, but was further evaluated in this study for confirmation or assignment of any missing functional features. The light grey sector indicates the total number of previously uncharacterized enzymes from JGI, for which putative functions were predicted based on UniProt annotations. The medium grey sector indicates the number of enzymes for which no annotations were available in JGI as well as in UniProt. A putative function for each of these enzymes was predicted using various bioinformatics tools. The values inside the chart refer to the total number of enzymes while the values outside the chart indicate the distribution of the enzymes per organism. The names of the fifteen microalgae are indicated in the right panel with different color codes for each species.

**Fig 10:**
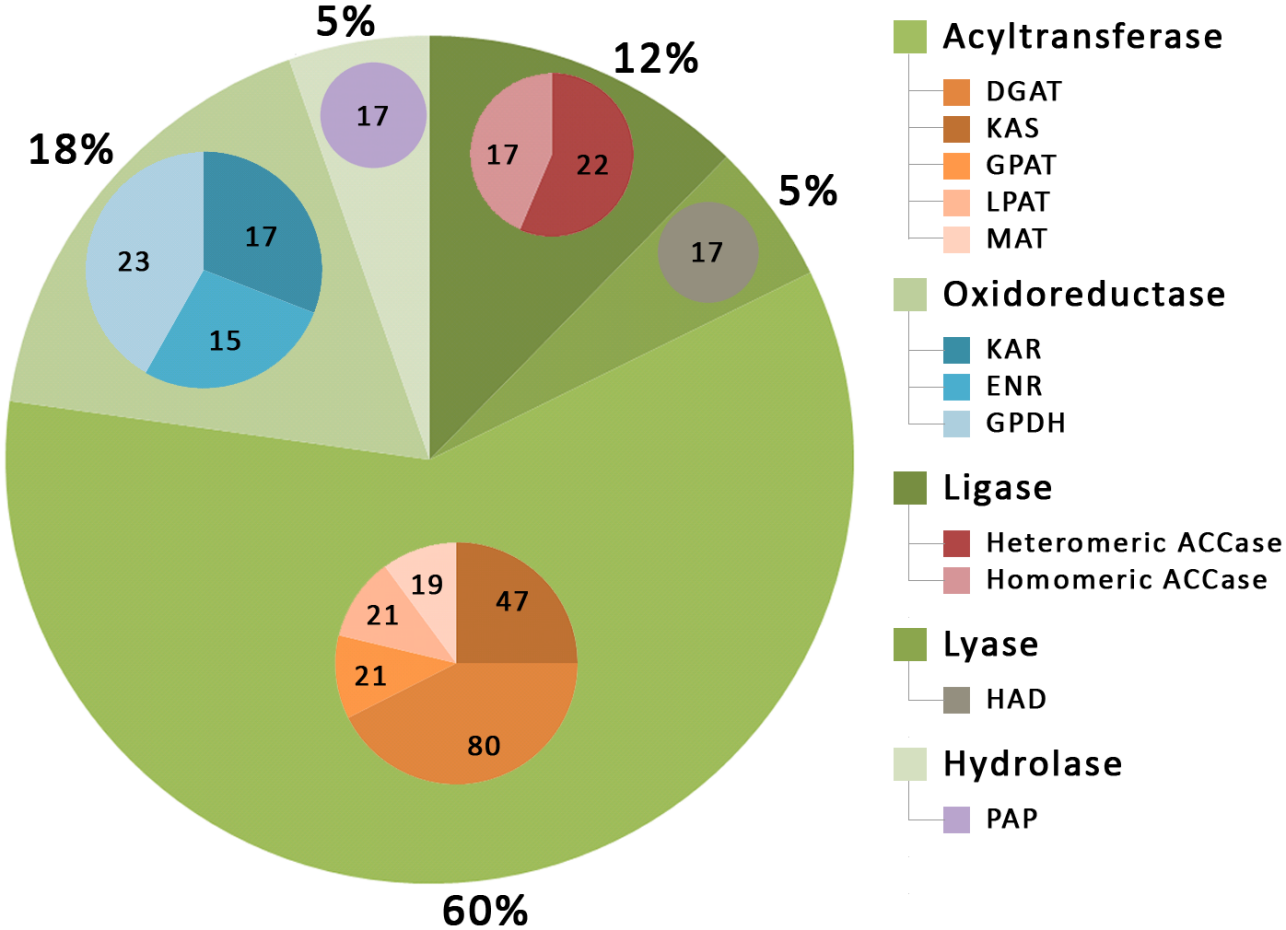
Distribution of sequences according to enzyme classification in the dEMBF database. The main chart shows the overall percentage of enzymes belonging to acyltransferase, oxidoreductase, ligase, lyase and hydrolase while the insert charts shows the total number of each enzymes (values indicated) belonging to a particular enzymes class.

After a thorough examination of the fifteen algal genomes, a total of 289 enzymes with putative roles in lipid synthesis were identified (S1 and S2 Tables). Sequence-structure information of these enzymes, together with the 27 well characterized homologous enzymes from Arabidopsis used as reference dataset in this study, are provided in the database. While previous studies have identified some key enzymes associated with lipid metabolic pathway in few algal species [41–49], the genomes of *C*. *variabilis*, *M*. *pusilla*, *Micromonas sp*., *B*. *prasinos*, *T*. *pseudonana*, *P*. *tricornutum*, *E*. *siliculosus*, *A*. *anophagefferens* and *E*. *huxleyi* have been mined for the first time in this study to collate the entire repertoire of enzymes responsible for lipid accumulation in microalgae. In addition to genome mining, we have assigned pathways, gene ontology terms and cluster of orthologous (S3 Table), subcellular location, secondary and tertiary structure, biophysical properties, cellular processes and protein family terms to each of the enzymes. Consequently, we have improved the existing functional annotation of all 289 enzymes including 86 previously uncharacterized sequences for which a putative function in lipid biosynthesis has been determined (Fig 9). We observed that the analyzed algal genomes exhibited an overall comparable enzymatic makeup and each encode the major enzymes for lipid synthesis similar to Arabidopsis (S2 Table and S1 Fig). However, we found that four algal species viz., *C*. *variabilis*. *C*. *reinhardtii*, *V*. *carteri and C*. *merolae* contain both homomeric and heteromeric ACCase enzyme, while the rest contain only the homomeric form of ACCase. This is in agreement to a previous published report, stating that the green (Chlorophyta) and red (Rhodophyta) algae with the exception of the green algal class Prasinophyceae (*O*. *lucimarinus*, *O*. *tauri*, *M*. *pusilla*, *Micromonas sp*. and *B*. *prasinos*) contain both homomeric and heteromeric ACCase while other algal species belonging to Heterokontophyta and Haptophyta lack heteromeric ACCase [42]. Furthermore, we found that the acyltransferases (60% of the total number of enzymes) is the most abundant enzyme class (Fig 10). The increased number of enzymes belonging to this class is probably significant considering that the three acyltransferases including GPAT, LPAT and DGAT catalyzes sequentially to acylate glycerol backbone, to ultimately produce TAG. These enzymes play a vital role in determining the acyl composition of glycerolipids and the final content of TAG [12]. In particular, relatively more number of DGAT (80 in number) followed by ACCase (39 in number) enzyme was observed in all algal genomes. The fact that ACCase catalyzes the initial rate limiting step of fatty acid biosynthesis by converting acetyl CoA to malonyl CoA while the DGAT enzyme drives the final step of TAG synthesis acylating diacylglycerol to TAG [4, 10], clearly reflects the high lipid accumulation capability of microalgae for biofuel production.

To our knowledge, dEMBF is the first comprehensive database on enzymes responsible for lipid accumulation in fifteen diverse algal species whose genome sequences are available. This work could be useful towards better understanding of fatty acid and TAG biosynthetic pathways in microalgae, besides facilitating the development of genetically engineered algal strains for a sustainable and economical viable biofuel production.

## Supporting Information

S1 Fig Genome-wide distribution of enzymes in the dEMBF database. Comparison of number of lipid biosynthesis enzymes in *Arabidopsis thaliana*, *Chlorella variabilis*, *Chlamydomonas reinhardtii*, *Ostreococcus lucimarinus*, *Ostreococcus tauri*, *Volvox carteri*, *Micromonas pusilla strain CCMP1545*, *Micromonas sp*. *strain RCC2999*, *Thalassiosira pseudonana*, *Phaeodactylum tricornutum*, *Ectocarpus siliculosus*, *Aureococcus anophagefferens*, *Cyanidioschyzon merolae*, *Emiliania huxleyi*, *Bathycoccus prasinos* and *Nannochloropsis gaditana*. Enzymes are indicated with different colors as defined in the legend.
(TIF)

S1 Table Distribution of enzymes (UniProt accession IDs) putatively involved in lipid biosynthesis in various algal species. (DOCX)

S2 Table Genome-wide comparative analysis of homologous lipid biosynthesis enzymes. (DOCX)

S3 Table Gene ontology classification and clusters of orthologous of lipid biosynthesis enzymes in the dEMBF database. (DOCX)
