# Supplementary figures and images for: dEMBF: A Comprehensive Database of Enzymes of Microalgal Biofuel Feedstock
Source: PLoS One. 2016 Jan 4;11(1):e0146158. doi: 10.1371/journal.pone.0146158 (PMC4699747; doi:10.1371/journal.pone.0146158)

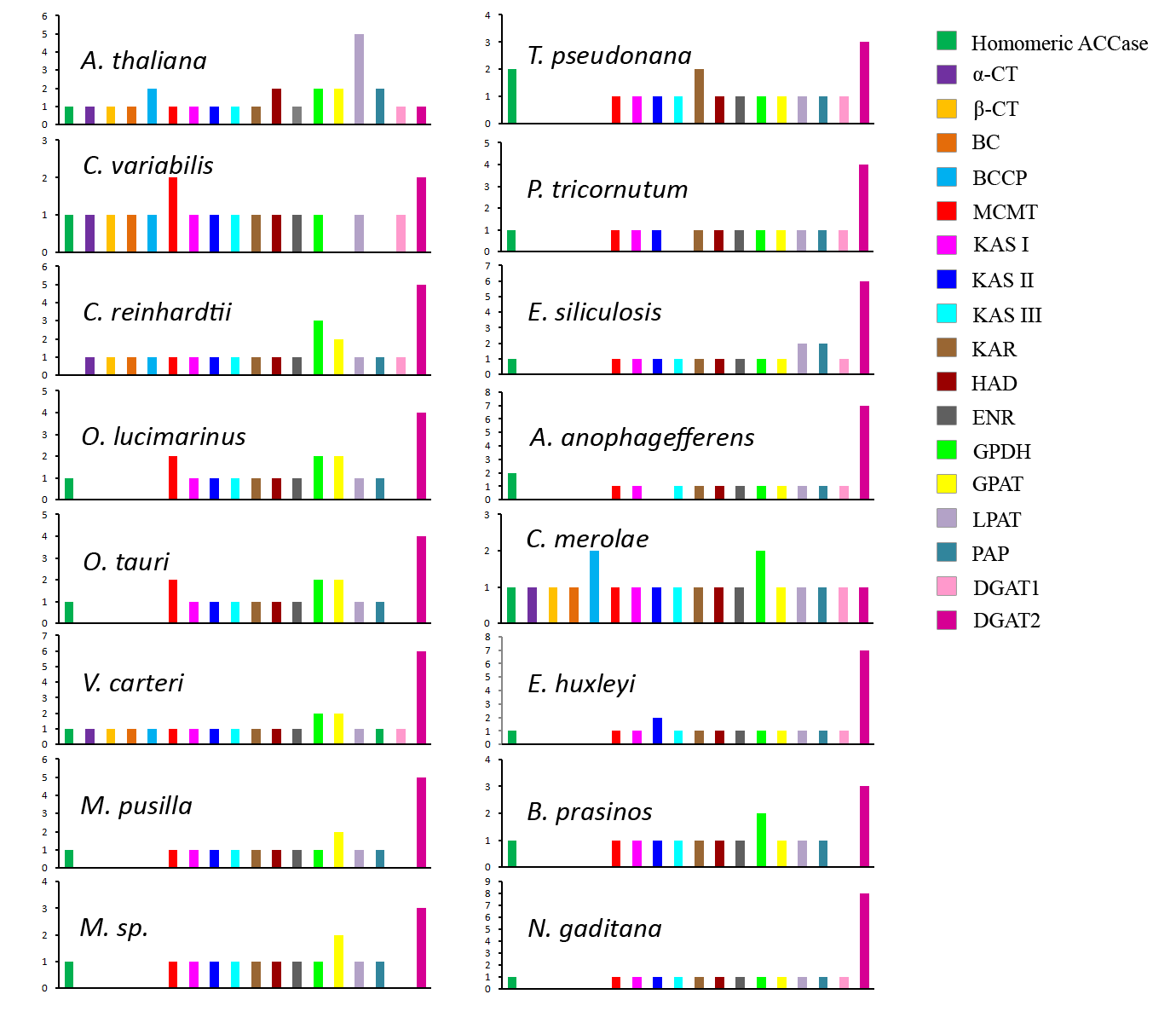

Supplement: S1 Fig — Comparison of number of lipid biosynthesis enzymes in Arabidopsis thaliana, Chlorella variabilis, Chlamydomonas reinhardtii, Ostreococcus lucimarinus, Ostreococcus tauri, Volvox carteri, Micromonas pusilla strain CCMP1545, Micromonas sp. strain RCC2999, Thalassiosira pseudonana, Phaeodactylum tricornutum, Ectocarpus siliculosus, Aureococcus anophagefferens, Cyanidioschyzon merolae, Emiliania huxleyi, Bathycoccus prasinos and Nannochloropsis gaditana. Enzymes are indicated with different colors as defined in the legend. (TIF) [file pone.0146158.s001.tif]
