# Supplementary material for: dEMBF: A Comprehensive Database of Enzymes of Microalgal Biofuel Feedstock
Source: PLoS One. 2016 Jan 4;11(1):e0146158. doi: 10.1371/journal.pone.0146158 (PMC4699747; doi:10.1371/journal.pone.0146158)
Supplement: S1 Table — (DOCX) [file pone.0146158.s002.docx]

**S1 Table .** Distribution of enzymes (UniProt accession IDs) putatively involved in lipid biosynthesis in various algal species ^a^.

| Microalgae | Hm  ACCase | α-CT | β-CT | BC | BCCP | MCMT | KAS I | KAS II | KAS III | KAR | HAD | ENR | GPDH | GPAT | LPAT | PAP | DGAT1 | DGAT2 |
| --- | --- | --- | --- | --- | --- | --- | --- | --- | --- | --- | --- | --- | --- | --- | --- | --- | --- | --- |
| *C. variabilis* | E1Z4T6^c^ | E1ZJK9^c^ | F2YGI4^c^ | E1Z5P4^c^ | E1Z723^c^ | E1Z713  E1ZCL0 | E1ZQQ3 | E1ZF89 | E1ZFD0 | E1ZFR4 | E1Z8J0 | E1Z2Y2 | E1ZFX7 |  | E1ZQN6 |  | E1ZTZ7 | E1ZSG9  E1ZP13 |
| *C. reinhardtii* |  | A8J646^b,c^ | A8JHU1^b,c^ | A8JGF4^b,c^ | A8JDA7^b,c^ | A8HP61^b^ | A8JEF7^b^ | A8JCK1^b^ | A8JHL7^b^ | Q84X75^b^ | A8IX17^b^ | A8JFI7^b^ | A8ICN2  A8IKS4  A8J130 | A8J0R2^b^  A8HVM5^b^ | A8J0J0^b^ | A8IK23^b^ | 323853^e,f^ | A8JGY1^e,f,g^  A8IAH9^e,f,g^  A8INZ7^e,f,g^  A8IXB2^b,e,f,g^  A8J110^e,f,g^ |
| *O. lucimarinus* | A4RRC3^b,c^ |  |  |  |  | A4S2U9^b^  A4SAC5^b^ | A4RSM2^b^ | A4S7B9^b^ | A4S7P4^b^ | A4RQY6^b^ | A4RUS8^b^ | A4S0L7^b^ | A4RRG  A4RWF3 | A4RT23^b^  A4S945^b^ | A4S0H0^b^ | A4RZP8^b^ |  | A4S872^b, e^  A4S1J0^e^  A4S6D6  A4S5J7 |
| *O. tauri* | Q01GA9^b^ |  |  |  |  | Q011G6^b^  Q00S12^b^ | A0A090M7I9^b^ | Q00V56^b^ | A0A096PB26^b^ | Q01GL3^b^ | A0A090LZ74 |  | Q01AJ0 | Q01F77^b^ | A0A090NV8^b^ | A0A090M7C8 |  | Q00UG1^b^  A0A090MCQ6^e,h^  A0A096P817  A0A090M8M4^e,h^ |
| *V. carteri* | D8UA31^b,c^ | D8TNY0^b,c^ | D8U455^b,c^ | D8UF54^b,c^ | D8U256^b,c^ | D8TTQ7^b^ | D8UDW0^b^ | D8TXC7 | D8TXF1^b^ | D8TK78^b^ | D8TV61^b^ | D8UC03^b^ | D8TPA  D8TPP4 | D8TVT7^b^  D8TIB3^b^ | D8U1V6^b^ | D8TNB7^b^ | D8UHL1^e^ | D8UGA9^b^  D8TSJ0^e^  D8TT47^e^  D8TS74^e^  D8TL45^e^  D8U2Q8^e^ |
| *M. pusilla* | C1ML75^c^ |  |  |  |  | C1MGN5 | C1MVN2 | C1MYJ6 | C1MYF8 | C1MKF9 | C1MJC2 | C1MZE1 | C1MXZ4 | C1MYL1  C1N800 | C1MQM1 | C1MRH2 |  | C1MMU7  C1MZ81  C1MZ66  C1N937  C1MRZ7 |
| *M. sp.* | C1FD95^c^ |  |  |  |  | C1FD34 | C1EC96 | C1EET5 | C1EEV4 | C1FDQ2 | C1DZI2 | C1E829 | C1EDN4 | C1EES2  C1E6V3 | C1DYE7 | C1E092 |  | C1E7X7  C1FIB5  C1E7W4 |
| *T. pseudonana* | B5YMF5^c^  B8BVD1^c^ |  |  |  |  | B8C2A9 | B8C1R1 | B8C5X7 | B8CGL6 | B8BTK3  B8BYY5 | B8C490 | B8BXA1 | B8BQG6 | B8C9T8 | B8CEH9 | B8BTF4 | B8BTU5^e^ | B8BR26  B8CEL8  B8BSU8 |
| *P. tricornutum* | B7G7S4^c^ |  |  |  |  | B7G3D4 | B7G2W2 | B7GCM0 |  | B7G1R8 | B7S3L6 | B7FS72 | B7FQ11 | B7GCP3 | B7FQL9 | B7FTF0 | B7FT04^e,i^ | B7FP00^e^  B7GAN7  I6QM80  B7FSB9 |
| *E. siliculosis* | D8LHP3^c^ |  |  |  |  | D8LN46 | D8LRQ2 | D8LLC3 | D8LRQ3 | D7FQ82 | D7FWP0 | D8LLF7 | D7FSV6 | D7FWA5 | D8LTF6  D8LS64 | D8LKT7  D7G275 | D8LSK0^e^ | D7G7D5^e^  D8LJK0^e^  D8LSD5^e^  D8LD78^e^  D8LL52^e^  D7FQF4^e^ |
| *A. anophagefferens* | F0YE78^c^  F0YJA4^c^ |  |  |  |  | F0YJC8 | F0Y6D5 |  | F0XWH7 | F0Y3V7 | F0YQ60 | F0XZA4 | F0YGY0 | F0YH70 | F0YPH1 | F0YKA3 | F0YLU4 | F0YG83  F0XZM2  F0Y2N5  F0XYV8  F0Y6W6  F0YDL5  F0YS11 |
| *C. merolae* | M1V8X5^c,d^ | Q85G50^c^ | Q85FS4^c^ | M1V7A2^c,d^ | Q85FY8^c^  M1VA64^c^ | M1VIH2^c,d^ | M1VIU1^d^ | M1UT44^d^ | M1UP45 | M1VHS0^d^ | M1VH29^d^ | M1UXU2^d^ | M1UP39  M1UWL0 | M1V7Y8^d^ | M1V4N2^d^ | M1VJ00 | M1UVP1^d^ | M1VCJ0 |
| *E. huxleyi* | R1FFT9^c^ |  |  |  |  | R1FRD8 | R1DM58 | R1D916  R1F3A0 | R1G8H9 | R1CUW5 | R1CAN9 | R1DPI9 | R1DBS0 | R1DEQ8 | R1E6C8 | R1FSH5 | R1EZX7 | R1DAJ2  R1D938  R1DEG2  R1BLZ1  R1G7X4  R1DRF3  R1DIW4 |
| *B. prasinos* | K8EIK1 |  |  |  |  | K8EIM2 | K8F4N5 | K8F005 | K8EAY0 | K8E9B9 | K8FIB1 | K8EU82 | K8EIV0  K8EY40 | K8FCP4 | K8EUG3 | K8F043 |  | K8F2W3  K8EQD6  K8F1N5 |
| *N. gaditana* | I2CQP5^j^ |  |  |  |  | S5VRZ9^j^ | W7TRD5^j^ | W7U686^j^ | I2CQW7^j^ | W7TF53^j^ | W7TUB8^j^ | I2CQQ0^j^ | W7TAY6^j^ | W7TUN5^j^ | W7U0D6^j^ | W7TQB3^j^ | W7TT63^j^ | W7TJC9^j^  W7T9Y9^j^  W7TQ66^j^  W7TT81^j^  W7T2V9^j^  W7THQ4^j^  W7TTN1^j^  W7U2A6^j^ |

Abbreviations: *C. variabilis- Chlorella variabilis, C. reinhardtii- Chlamydomonas reinhardtii, O. lucimarinus- Ostreococcus lucimarinus, O. tauri- Ostreococcus tauri, V. carteri- Volvox carteri, M. pusilla- Micromonas pusilla strain CCMP1545, M. sp.- Micromonas sp. strain RCC2999, T. pseudonana- Thalassiosira pseudonana, P. tricornutum- Phaeodactylum tricornutum, E. siliculosus- Ectocarpus siliculosus, A. anophagefferens- Aureococcus anophagefferens, C. merolae- Cyanidioschyzon merolae, E. huxleyi- Emiliania huxleyi, B. prasinos- Bathycoccus prasinos, N. gaditana- Nannochloropsis gaditana*, Hm ACCase - Homomeric Acetyl-CoA carboxylase, α-CT- Acetyl-CoA carboxylase alpha-carboxyltransferase, β-CT- Acetyl-CoA carboxylase beta-carboxyltransferase, BC- Biotin carboxylase, BCCP- Biotin carboxyl carrier protein, MCMT- Malonyl-CoA-ACP Malonyltransacylase, KAS I- beta-ketoacyl-ACP Synthase I, , KAS II- beta-ketoacyl-ACP Synthase II, , KAS III- beta-ketoacyl-ACP Synthase III, KAR- 3-ketoacyl-ACP Reductase, HAD- 3-hydroxyacyl-ACP Dehydratase, ENR- Enoyl-ACP Reductase, GPDH- NAD-dependent Glycerol-3-phosphate dehydrogenase, GPAT- Glycerol-3-phosphate acyltransferase, LPAT- Lysophosphatidyl acyltransferase, PAP- Phosphatidate phosphatase, DGAT1- Diacylglycerol acyltransferase Type 1, DGAT2- Diacylglycerol acyltransferase Type 2.

^a^ Arabidopsis enzymes experimentally validated to be involved in lipid biosynthesis (see Table 1) were used as a BLASTp query against the aforementioned 15 sequenced algal species and hits with less than 1E-5 were designated as homologs. UniProt IDs are unique for each species. Enzyme ID numbers that are previously identified by sequence similarity and have been reported in literature are marked with relevant references. Enzyme ID numbers underlined indicate proteins that have been biochemically characterised.

^b^ See Reference 41.

^c^ See Reference 42.

^d^ See Reference 43.

^e^ See Reference 44.

^f^ See Reference 45.

^g^ See Reference 46.

^h^ See Reference 47.

^i^ See Reference 48.

^j^ See Reference 49.
