# Supplementary material for: dEMBF: A Comprehensive Database of Enzymes of Microalgal Biofuel Feedstock
Source: PLoS One. 2016 Jan 4;11(1):e0146158. doi: 10.1371/journal.pone.0146158 (PMC4699747; doi:10.1371/journal.pone.0146158)
Supplement: S2 Table — (DOCX) [file pone.0146158.s003.docx]

**S2 Table.**  Genome-wide comparative analysis of homologous lipid biosynthesis enzymes.

|  | Streptophyta | Chlorophyta | | | | | | | | Heterokontophyta | | | | | Rhodophyta | Haptophyta |  |
| --- | --- | --- | --- | --- | --- | --- | --- | --- | --- | --- | --- | --- | --- | --- | --- | --- | --- |
| Enzyme name | *At* | *Cv* | *Cr* | *Ol* | *Ot* | *Vc* | *Mp* | *Msp.* | *Bp* | *Tp* | *Pt* | *Es* | *Aa* | *Ng* | *Cm* | *Eh* | **Total**^a^ |
| Fatty acid biosynthesis^b^ | | | | | | | | | | | | | | | | |  |
| Homomeric ACCase | 1 | 1 | 0 | 1 | 1 | 1 | 1 | 1 | 1 | 2 | 1 | 1 | 2 | 1 | 1 | 1 | 17 |
| alpha-CT | 1 | 1 | 1 | 0 | 0 | 1 | 0 | 0 | 0 | 0 | 0 | 0 | 0 | 0 | 1 | 0 | 5 |
| beta-CT | 1 | 1 | 1 | 0 | 0 | 1 | 0 | 0 | 0 | 0 | 0 | 0 | 0 | 0 | 1 | 0 | 5 |
| BC | 1 | 1 | 1 | 0 | 0 | 1 | 0 | 0 | 0 | 0 | 0 | 0 | 0 | 0 | 1 | 0 | 5 |
| BCCP | 2 | 1 | 1 | 0 | 2 | 1 | 0 | 0 | 0 | 0 | 0 | 0 | 0 | 0 | 2 | 0 | 7 |
| MCMT | 1 | 2 | 1 | 2 | 2 | 1 | 1 | 1 | 1 | 1 | 1 | 1 | 1 | 1 | 1 | 1 | 19 |
| KAS I | 1 | 1 | 1 | 1 | 1 | 1 | 1 | 1 | 1 | 1 | 1 | 1 | 1 | 1 | 1 | 1 | 16 |
| KAS II | 1 | 1 | 1 | 1 | 1 | 1 | 1 | 1 | 1 | 1 | 1 | 1 | 0 | 1 | 1 | 2 | 16 |
| KAS III | 1 | 1 | 1 | 1 | 1 | 1 | 1 | 1 | 1 | 1 | 0 | 1 | 1 | 1 | 1 | 1 | 15 |
| KAR | 1 | 1 | 1 | 1 | 1 | 1 | 1 | 1 | 1 | 2 | 1 | 1 | 1 | 1 | 1 | 1 | 17 |
| HAD | 2 | 1 | 1 | 1 | 1 | 1 | 1 | 1 | 1 | 1 | 1 | 1 | 1 | 1 | 1 | 1 | 17 |
| ENR | 1 | 1 | 1 | 1 | 0 | 1 | 1 | 1 | 1 | 1 | 1 | 1 | 1 | 1 | 1 | 1 | 15 |
| Triacylglycerol (TAG) assembly^b^ | | | | | | | | | | | | | | | | |  |
| GPDH | 2 | 1 | 3 | 2 | 1 | 2 | 1 | 1 | 2 | 1 | 1 | 1 | 1 | 1 | 2 | 1 | 23 |
| GPAT | 2 | 0 | 2 | 2 | 1 | 2 | 2 | 2 | 1 | 1 | 1 | 1 | 1 | 1 | 1 | 1 | 21 |
| LPAT | 5 | 1 | 1 | 1 | 1 | 1 | 1 | 1 | 1 | 1 | 1 | 2 | 1 | 1 | 1 | 1 | 21 |
| PAP | 2 | 0 | 1 | 1 | 1 | 1 | 1 | 1 | 1 | 1 | 1 | 2 | 1 | 1 | 1 | 1 | 17 |
| DGAT 1 | 1 | 1 | 1 | 0 | 0 | 1 | 0 | 0 | 0 | 1 | 1 | 1 | 1 | 1 | 1 | 1 | 11 |
| DGAT 2 | 1 | 2 | 5 | 4 | 4 | 6 | 5 | 3 | 3 | 3 | 4 | 6 | 7 | 8 | 1 | 7 | 69 |
| **Total**^c^ | 27 | 18 | 24 | 19 | 16 | 25 | 18 | 16 | 16 | 18 | 16 | 21 | 20 | 21 | 20 | 21 | **316** |

Abbreviations: At- *Arabidopsis thaliana*,  *Cv- Chlorella variabilis, Cr- Chlamydomonas reinhardtii, Ol- Ostreococcus lucimarinus, Ot- Ostreococcus tauri, V. carteri- Volvox carteri, M. pusilla- Micromonas pusilla strain CCMP1545, M. sp.- Micromonas sp. strain RCC2999, B. prasinos- Bathycoccus prasinos, T. pseudonana- Thalassiosira pseudonana, P. tricornutum- Phaeodactylum tricornutum, E. siliculosus- Ectocarpus siliculosus, A. anophagefferens- Aureococcus anophagefferens, N. gaditana- Nannochloropsis gaditana C. merolae- Cyanidioschyzon merolae, E. huxleyi- Emiliania huxleyi*. Homomeric ACCase-Homomeric Acetyl-CoA carboxylase, alpha-CT- Acetyl-CoA carboxylase alpha-carboxyltransferase, beta-CT- Acetyl-CoA carboxylase beta-carboxyltransferase, BC- Biotin carboxylase, BCCP- Biotin carboxyl carrier protein, MCMT- Malonyl-CoA-ACP Malonyltransacylase, KAS I- beta-ketoacyl-ACP Synthase I, , KAS II- beta-ketoacyl-ACP Synthase II, , KAS III- beta-ketoacyl-ACP Synthase III, KAR- 3-ketoacyl-ACP Reductase, HAD- 3-hydroxyacyl-ACP Dehydratase, ENR- Enoyl-ACP Reductase, GPDH- NAD-dependent Glycerol-3-phosphate dehydrogenase, GPAT- Glycerol-3-phosphate acyltransferase, LPAT- Lysophosphatidyl acyltransferase, PAP- Phosphatidate phosphatase, DGAT 1- Diacylglycerol acyltransferase Type 1, DGAT 2- Diacylglycerol acyltransferase Type 2.

^a^ Categories of lipid biosynthetic enzymes, sorted by fatty acid biosynthesis an TAG assembly.

^b^ Total number of enzymes belonging to a particular enzyme category.

^c^ Total number of enzymes in an organism.
