# Supplementary material for: dEMBF: A Comprehensive Database of Enzymes of Microalgal Biofuel Feedstock
Source: PLoS One. 2016 Jan 4;11(1):e0146158. doi: 10.1371/journal.pone.0146158 (PMC4699747; doi:10.1371/journal.pone.0146158)
Supplement: S3 Table — (DOCX) [file pone.0146158.s004.docx]

**S3 Table .** Gene ontology classification and clusters of orthologous of lipid biosynthesis enzymes in the dEMBF database.

| **Enzyme name** | **GO-term^a^** | **GO-term description^b^** | **KOG ID^c^** | **KEGG Orthology** | **OrthoMCL Group ID^d^** |
| --- | --- | --- | --- | --- | --- |
| **GO:0006633 Fatty acid biosynthetic process** | | | | | |
|  | **GO:0016874** | **Ligase activity** |  |  |  |
| Homomeric ACCase | GO:0003989 | acetyl-CoA carboxylase activity | KOG0238 | K01961 | OG5_126626 |
| alpha-CT | GO:0003989 | acetyl-CoA carboxylase activity | - | K01962 | OG5_132080 |
| beta-CT | GO:0003989 | acetyl-CoA carboxylase activity | KOG0540 | K01963 | OG5_132573 |
| BC | GO:0003989 | acetyl-CoA carboxylase activity | KOG0238 | K01961 | OG5_126626 |
| BCCP | GO:0003989 | acetyl-CoA carboxylase activity | - | K02160 | OG5_131515 |
|  | **GO:0016836** | **Hydro-lyase activity** |  |  |  |
| HAD | GO:0019171 | 3-hydroxyacyl-[acyl-carrier-protein] dehydratase |  | K02372 | OG5_130367 |
|  | **GO:0016614** | **Oxidoreductase activity** |  |  |  |
| KAR | GO:0004316 | 3-oxoacyl-[acyl-carrier-protein] reductase (NADPH) | KOG1200 | K00059 | OG5_126618 |
| ENR | GO:0016631 | enoyl-[acyl-carrier-protein] reductase | KOG0725 | K00208 | OG5_130466 |
|  | **GO:0016740** | **Transferase activity** |  |  |  |
| KAS I | GO:0004315 | 3-oxoacyl-[acyl-carrier-protein] synthase | KOG1394 | K09458 | OG5_126985 |
| KAS II | GO:0004315 | 3-oxoacyl-[acyl-carrier-protein] synthase | KOG1394 | K09458 | OG5_126985 |
| KAS III | GO:0004315 | 3-oxoacyl-[acyl-carrier-protein] synthase | KOG1390 | K00648 | OG5_129509 |
| MCMT | GO:0004314 | [acyl-carrier-protein] S-malonyltransferase | KOG2926 | K00645 | OG5_127857 |
| **GO:0019432 Triglyceride (TAG) biosynthetic process** | | | | | |
|  | **GO:0016740** | **Transferase activity** |  |  |  |
| GPAT | GO:0004366 | glycerol-3-phosphate O-acyltransferase | KOG2898 | K00630 | OG5_137532 |
| LPAT | GO:0003841 | 1-acylglycerol-3-phosphate O-acyltransferase | KOG2848 | K13523 | OG5_126904 |
| DGAT 1 | GO:0004144 | diacylglycerol O-acyltransferase | KOG0380 | K11155 | OG5_128200 |
| DGAT 1 | GO:0004144 | diacylglycerol O-acyltransferase | KOG0831 | K14457 | OG5_127120 |
|  | **GO:0004553** | **Hydrolase activity** |  |  |  |
| PAP | GO:0008195 | phosphatidate phosphatase | KOG2116 | K15728 | OG5_127287 |
|  | **GO:0016614** | **Oxidoreductase activity** |  |  |  |
| GPDH | GO:0004367 | glycerol-3-phosphate dehydrogenase [NAD+] | KOG2711 | K00006 | OG5_126704 |

Abbreviations: Homomeric ACCase-Homomeric Acetyl-CoA carboxylase, alpha-CT- Acetyl-CoA carboxylase alpha-carboxyltransferase, beta-CT- Acetyl-CoA carboxylase beta-carboxyltransferase, BC- Biotin carboxylase, BCCP- Biotin carboxyl carrier protein, MCMT- Malonyl-CoA-ACP Malonyltransacylase, KAS I- beta-ketoacyl-ACP Synthase I, , KAS II- beta-ketoacyl-ACP Synthase II, , KAS III- beta-ketoacyl-ACP Synthase III, KAR- 3-ketoacyl-ACP Reductase, HAD- 3-hydroxyacyl-ACP Dehydratase, ENR- Enoyl-ACP Reductase, GPDH- NAD-dependent Glycerol-3-phosphate dehydrogenase, GPAT- Glycerol-3-phosphate acyltransferase, LPAT- Lysophosphatidyl acyltransferase, PAP- Phosphatidate phosphatase, DGAT 1- Diacylglycerol acyltransferase Type 1, DGAT 2- Diacylglycerol acyltransferase Type 2.

^a^ Gene Ontology term as predicted using AmiGO.

^b^ Gene Ontology term description.

^c^ Cluster of Orthologous group determined using KOGnitor.

^d^ Indicates Orthologous group ID as predicted using OrthoMCL, to which that gene belongs to.
